# Structural basis for the synaptic adhesion protein ELFN1-mediated modulation of mGlu7

**DOI:** 10.1093/procel/pwag034

**Published:** 2026-05-22

**Authors:** Shuling Lin, Ye Feng, Shanhui Shui, Mengmeng Chen, Xiaojing Chu, Cuiying Yi, Shuo Han, Qiang Zhao, Beili Wu

**Affiliations:** State Key Laboratory of Drug Research, State Key Laboratory of Chemical Biology, Shanghai Institute of Materia Medica, Chinese Academy of Sciences, Shanghai 201203, China; School of Life Science and Technology, ShanghaiTech University, Shanghai 201210, China; State Key Laboratory of Drug Research, State Key Laboratory of Chemical Biology, Shanghai Institute of Materia Medica, Chinese Academy of Sciences, Shanghai 201203, China; University of Chinese Academy of Sciences, Beijing 100049, China; School of Pharmaceutical Science and Technology, Hangzhou Institute for Advanced Study, University of Chinese Academy of Sciences, Hangzhou 310018, China; State Key Laboratory of Drug Research, State Key Laboratory of Chemical Biology, Shanghai Institute of Materia Medica, Chinese Academy of Sciences, Shanghai 201203, China; State Key Laboratory of Drug Research, State Key Laboratory of Chemical Biology, Shanghai Institute of Materia Medica, Chinese Academy of Sciences, Shanghai 201203, China; State Key Laboratory of Drug Research, State Key Laboratory of Chemical Biology, Shanghai Institute of Materia Medica, Chinese Academy of Sciences, Shanghai 201203, China; University of Chinese Academy of Sciences, Beijing 100049, China; State Key Laboratory of Drug Research, State Key Laboratory of Chemical Biology, Shanghai Institute of Materia Medica, Chinese Academy of Sciences, Shanghai 201203, China; University of Chinese Academy of Sciences, Beijing 100049, China; Zhongshan Institute for Drug Discovery, Shanghai Institute of Materia Medica, Chinese Academy of Sciences, Zhongshan 528449, China; State Key Laboratory of Drug Research, State Key Laboratory of Chemical Biology, Shanghai Institute of Materia Medica, Chinese Academy of Sciences, Shanghai 201203, China; School of Life Science and Technology, ShanghaiTech University, Shanghai 201210, China; University of Chinese Academy of Sciences, Beijing 100049, China; School of Pharmaceutical Science and Technology, Hangzhou Institute for Advanced Study, University of Chinese Academy of Sciences, Hangzhou 310018, China; Shanghai Academy of Natural Sciences (SANS), Shanghai Institute of Materia Medica, Chinese Academy of Sciences, Shanghai 201203, China

**Dear Editor,**

Precise spatial and temporal organization of neurotransmitter receptors are essential to modulate the neurotransmitter release machinery (Tang et al., 2016), which is fundamental to the meticulous coordination of neurotransmission (de Wit and Ghosh, 2016). The metabotropic glutamate receptors (mGlus) are critical neurotransmitter receptors that fine tune excitatory and inhibitory neurotransmission across synapses (Niswender and Conn, 2010). Extracellular leucine-rich repeat and fibronectin type-III domain-containing protein 1 (ELFN1), a synaptic adhesion protein (Dolan et al., 2007), has been reported to participate in the recruitment, organization and functional modulation of group III mGlus such as mGlu4, mGlu6, and mGlu7 (Dunn et al., 2018; Matsunaga and Aruga, 2021; Stachniak et al., 2019). Deficiency of the mGlu-ELFN1 interaction leads to severe pathological outcomes such as epilepsy and night blindness (Matsunaga and Aruga, 2021). In addition, ELFN1 also serves as an endogenous allosteric modulator of group III mGlus (Dunn et al., 2018). However, previous findings exhibit discrepancies. The ELFN1 ectodomain fusion protein induced constitutive activation of mGlu7 (Stachniak et al., 2019), while the full-length ELFN1 decreased the maximal agonist response of this receptor (Lei et al., 2025). These discrepancies potentially result from differential modulation patterns under different ELFN1 concentrations and/or the membrane anchorage of the full-length ELFN1 that impose steric constraints. These studies suggest complexity and diversity of the ELFN1-mediated modulation of the mGlus, but the underlying mechanisms remain elusive. To better understand the transsynaptic recruitment and functional modulation between ELFN1 and mGlus, we determined the cryo-electronic microscopy (cryo-EM) structures of mGlu7 bound to the ELFN1 ectodomain (ELFN1^ECD^) in five different conformations and performed extensive functional assays to study the ELFN1-mediated modulation of mGlu7.

To enable structure determination of the ELFN1-bound mGlu7, the extracellular domain (ECD) of ELFN1 (residues D28–Y418) and the full-length mGlu7 were expressed individually and then assembled *in vitro*. The purified complex protein was subjected to cryo-EM single-particle analysis, which yielded five maps of the complex at 3.1–4.0 Å resolutions (Figs. 1A, 1B and S1; Table S1). The maps allowed unambiguous modeling of the Venus flytrap domains (VFTs) and partial residues of the cysteine-rich domains (CRDs) in the mGlu7 homodimer and the leucine rich-repeat (LRR) domain of ELFN1, but the transmembrane domains (TMDs) of the receptor were not resolved, indicating high dynamics in this region (Figs. 1A, 1B and S2).

**Figure 1.**
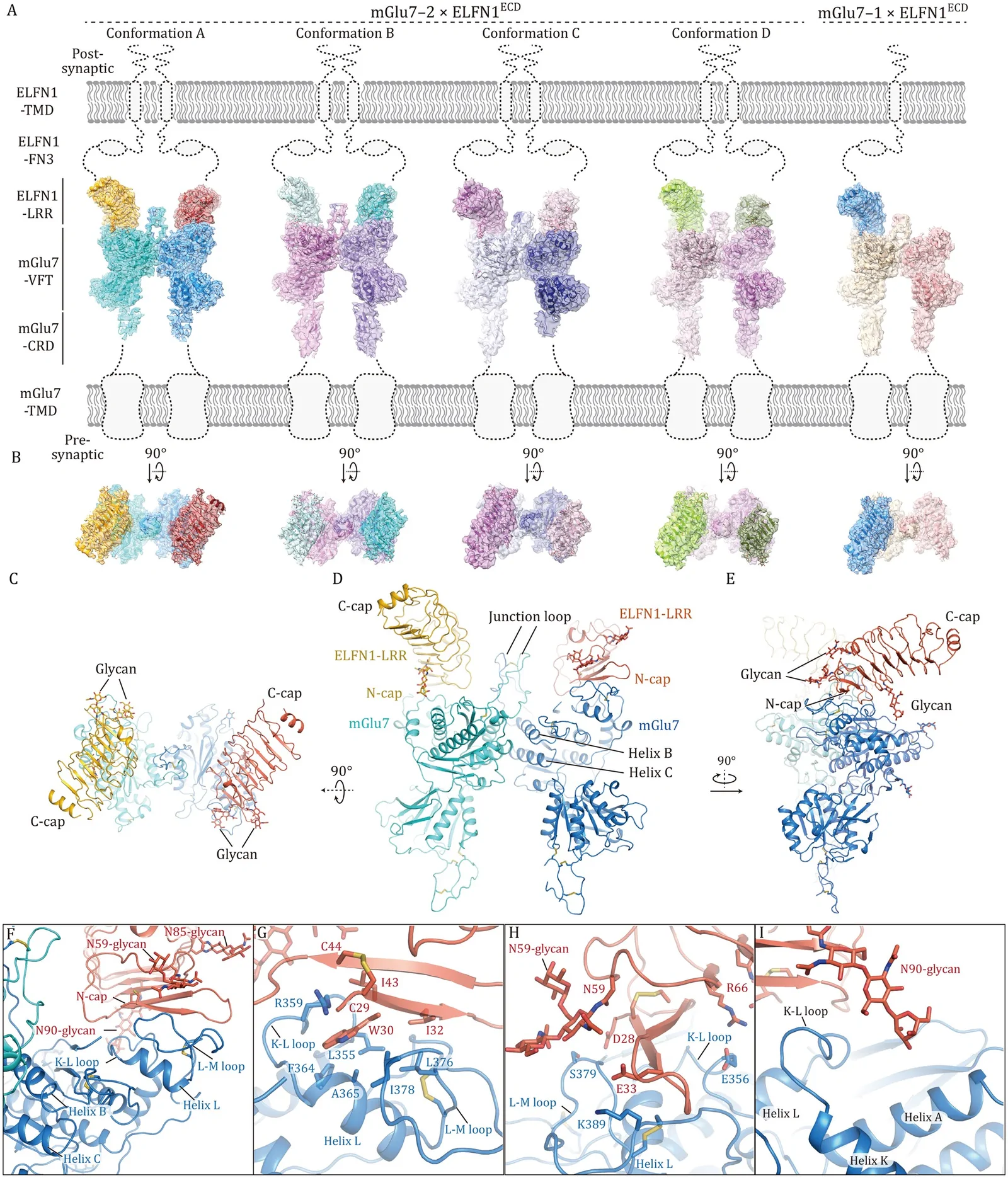
**Cryo-EM maps and overall structures of the mGlu7–ELFN1^ECD^ complexes and interactions between mGlu7 and ELFN1^ECD^**. (A and B) Cryo-EM maps and structures of the mGlu7–2 × ELFN1^ECD^ (conformations A to D) and mGlu7–1 × ELFN1^ECD^ complexes in membrane view (A) and extracellular view (B). The maps and structures are colored according to chains. The glycosylation sites and glycans are shown as sticks. The cell membranes are indicated by schematics of lipid bilayers. The missing regions of mGlu7 and ELFN1 are shown as dashed lines. (C–E) Structure of the mGlu7–2 × ELFN1^ECD^ complex in conformation A. The structure is shown in extracellular view (C) and membrane view in two orientations (D and E). C-cap, C-terminal cap; N-cap, N-terminal cap. (F) Overall view of the binding mode between ELFN1^ECD^ and mGlu7 in the mGlu7–2 × ELFN1^ECD^ complex (conformation A). (G) Enlarged view showing the hydrophobic interactions between ELFN1^ECD^ and mGlu7. (H) Enlarged view showing the hydrophilic interactions between ELFN1^ECD^ and mGlu7. (I) The interaction between the glycan branched at the ELFN1 residue N90 and mGlu7.

Four of the five structures exhibit a 2:2 (mGlu7 subunit: ELFN1^ECD^) stoichiometry of the complex (mGlu7–2 × ELFN1^ECD^), with one ELFN1 molecule bound to the apex of the VFT in each mGlu7 subunit, while in the other structure, the receptor couples to only one ELFN1 molecule with one of its subunits (2:1 stoichiometry; mGlu7–1 × ELFN1^ECD^) (Fig. 1A and 1B). The receptors in the five mGlu7–ELFN1^ECD^ structures display a similar overall architecture but are slightly different in the relative orientation between the two receptor subunits (Fig. 1A and 1B). In these structures, the VFTs of mGlu7 adopt an open conformation with helices B and C and the junction loop between helix B and β-sheet d in their top lobes mediating the dimerization (Fig. 1A and 1D). The conformation of the ELFN1^ECD^-bound VFTs is similar to that in our previously determined structure of inactive mGlu7 (PDB ID: 7EPC) (Du et al., 2021), but is more compact, with the bottom lobes of the VFTs in closer proximity (Fig. S3A–D). The Cα root mean square deviation (RMSD) between the ECDs in the inactive mGlu7 structure and either of the mGlu7–ELFN1^ECD^ structures is 1.3–2.3 Å, which suggests conformational heterogeneity and dynamics of the receptor upon ELFN1 binding. According to the RMSD values, the four mGlu7–2 × ELFN1^ECD^ structures are termed conformations A-D, with conformation A exhibiting the highest value (2.3 Å) and conformation D having the lowest (1.3 Å). In contrast to the diverse receptor conformations of the mGlu7–2 × ELFN1^ECD^ complex, a single conformation was observed for the receptor upon binding to only one ELFN1 molecule (Cα RMSD compared to the inactive mGlu7, 1.8 Å) (Fig. 1A and 1B). This difference suggests differential modulation modes that may correlate with the stoichiometry of the complex under different local ELFN1 concentrations or different states of receptor activation, which however, requires further evidence for full understanding.

In both the mGlu7–1 × ELFN1^ECD^ and mGlu7–2 × ELFN1^ECD^ complex structures, the LRR domain of ELFN1 adopts a classical horseshoe-shaped solenoid structure like those in most LRR-containing proteins (Dolan et al., 2007) (Fig. S3E). However, unlike other LRR-containing proteins, in which the LRR domains bind protein partners through their concave, convex and/or lateral surfaces (Jackson et al., 2016; Ranaivoson et al., 2015), the LRR domain of ELFN1 stacks on top of the mGlu7 VFT with its N-terminal cap forming an extended β-hairpin and attaching to the apex of the top lobe in mGlu7 (Figs. 1C–E and S3F). In the mGlu7–2 × ELFN1^ECD^ complexes, the two ELFN1 molecules adopt a similar binding pattern to the mGlu7 subunits, but display opposite binding orientations due to the symmetry of the receptor dimer (Fig. 1C–E).

The mGlu7-ELFN1 binding is mainly mediated through interactions between helix L and the connecting loops at its N and C termini (K-L loop and L-M loop) on the apex of each mGlu7 VFT and the N-terminal tip of ELFN1 (Figs. 1F and S3G). The side chain of the bulky residue W30 on the concave tip of ELFN1 inserts into a binding crevice on the mGlu7 apex, with a hydrophobic core composed of the receptor residues L355, F364, A365, and I378 on one side and the basic residue R359 on the opposite side that forms a π-cation interaction with the indole ring of W30 (Fig. 1G). The interactions with the concave region of ELFN1-LRR also involve a hydrophobic interaction network formed by the ELFN1 residues I32 and I43 and the mGlu7 residues L355 and L376, as well as an ionic interaction between R66 in ELFN1 and E356 in the K-L loop of the receptor (Fig. 1G and 1H). On the convex surface of ELFN1-LRR, D28 and E33 are within interaction distance of the residues S379 and K389 in the L-M loop of mGlu7, respectively (Fig. 1H). This aligns with a previous finding that the N-terminal residue D28 of ELFN1 is essential for mGlu6 binding (Dunn et al., 2018).

Supported by strong electron densities in the cryo-EM maps, N-glycosylation was observed at the residues N59, N85, and N90 of ELFN1 in all the five ELFN1^ECD^-bound mGlu7 structures (Figs. 1F and S2). Notably, the glycans branched at the residue N59 on the convex surface of the ELFN1-LRR domain and N90 on the concave surface approach the apical surface of the mGlu7 VFT and likely form additional interactions to further strengthen the mGlu7-ELFN1 binding (Fig. 1F, 1H and 1I). The N59-associated glycans lean against the L-M loop, while the glycans at N90 penetrate into a shallow groove formed by helix A and K-L loop in the receptor VFT (Fig. 1H and 1I).

To characterize the effect of ELFN1 on the receptor conformational rearrangement, we monitored the glutamate-induced inter-subunit conformational changes of the mGlu7 VFTs using a cell-based time-resolved fluorescence resonance energy transfer (trFRET) assay (Doumazane et al., 2013) (Fig. S4A). The data show that glutamate stimulates a dose-dependent decrease of the FRET efficiency for the wild-type mGlu7, which is associated with an increased distance between the fluorophores labelled at the N termini of the two mGlu7 subunits upon receptor activation (Fig. S4B). Addition of the purified wild-type ELFN1^ECD^ protein at a final concentration of 0.7 μmol/L further promoted the glutamate-induced reduction of trFRET signal by decreasing the FRET signal measured at the highest glutamate concentration (FRET_h_) by about 35% (Figs. 2A and S4B; Table S2). These data suggest that ELFN1^ECD^ has a positive effect on the receptor conformational rearrangement upon activation. To assess the importance of the mGlu7-ELFN1 interactions in mediating the modulation of ELFN1 on the receptor activation, the conformational changes of the mGlu7 VFTs were also measured for the wild-type receptor in the presence of the ELFN1^ECD^ proteins carrying the alanine mutation of D28, W30, I32, E33, I43, or R66. As expected, these mutants exhibited limited effects on the glutamate-induced reduction of the trFRET signal (Figs. 2A, S4B and S4C; Table S2).

**Figure 2.**
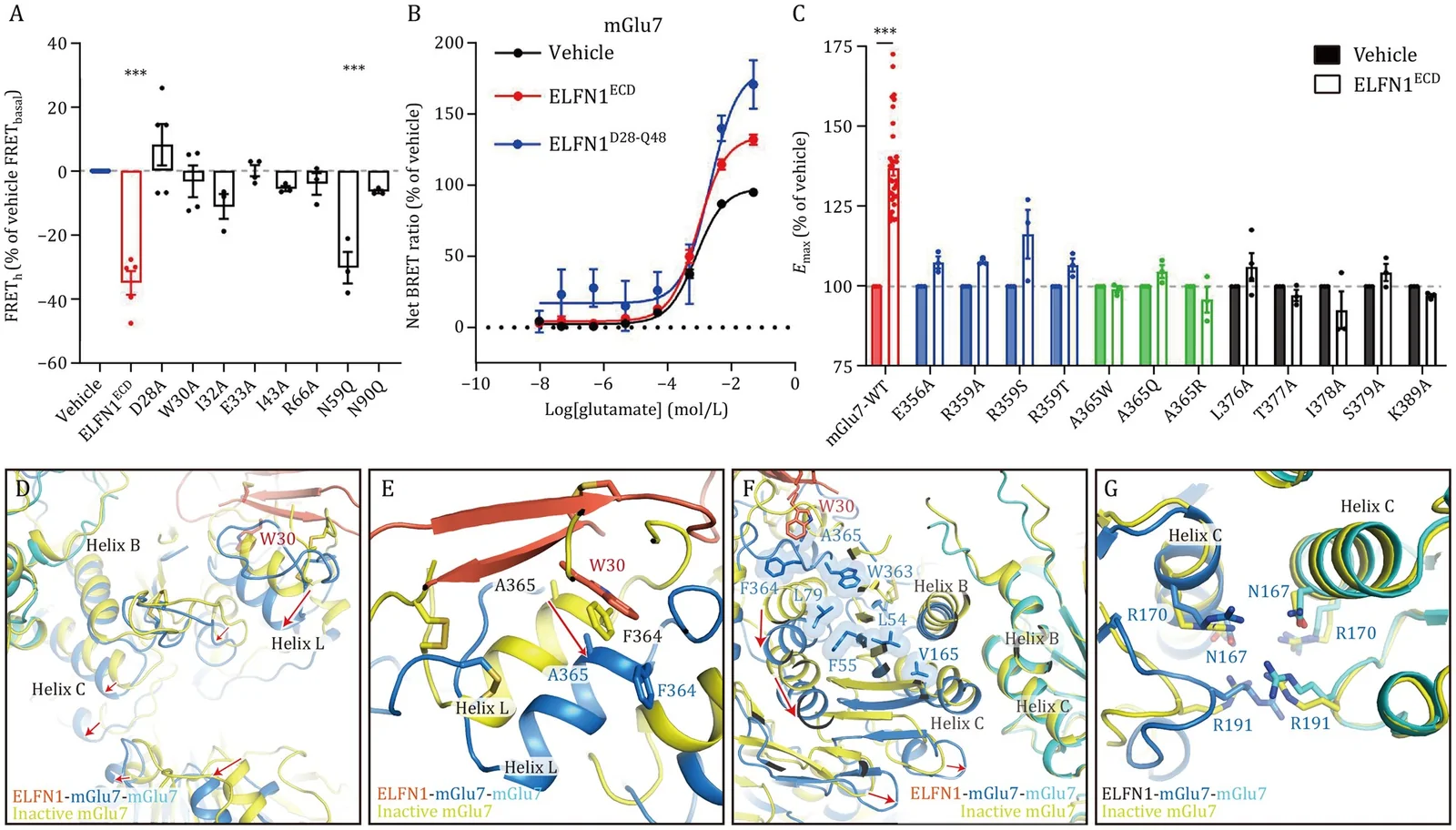
**ELFN1^ECD^-mediated modulation of mGlu7**. (A) The trFRET signal of mGlu7 supplemented with the wild-type ELFN1^ECD^ protein and mutants was measured at the highest glutamate concentration. Data are shown as mean ± SEM from at least three independent experiments performed in technical triplicate. ***P < 0.0001 by one-way analysis of variance followed by Dunnett’s post-test compared to the response of the vehicle group. Table S2 provides detailed numbers of independent experiments (*n*) and statistical evaluation. (B) Glutamate-induced G_i_ activation of mGlu7 in the presence or absence (vehicle) of ELFN1^ECD^ or 10 µmol/L ELFN1^D28-Q48^ peptide. Data are shown as mean ± SEM from at least three independent experiments performed in technical duplicate. Table S3 provides detailed numbers of independent experiments (*n*) and statistical evaluation. (C) The maximal response (*E*_max_) of the glutamate-induced G_i_ activation of the wild-type mGlu7 and the mutants within the ELFN1 binding site in the absence or presence of ELFN1^ECD^. The *E*_max_ measured in the presence of ELFN1^ECD^ is normalized to each vehicle group (ELFN1^ECD^-free). Data are shown as mean ± SEM from at least three independent experiments performed in technical duplicate. ***P < 0.0001 by one-way analysis of variance followed by Dunnett’s post-test compared to the response of each vehicle group. Table S3 provides detailed numbers of independent experiments (*n*) and statistical evaluation. (D) Structural comparison of the mGlu7–2 × ELFN1^ECD^ complex (conformation A) and the ELFN1-free, inactive mGlu7 (PDB ID: 7EPC). The red arrows indicate the movement of the mGlu7 VFT in the ELFN1-bound structure relative to the ELFN1-free structure. (E) ELFN1^ECD^-induced movement of helix L in the mGlu7 VFT, as indicated by a red arrow. (F) Hydrophobic interaction network involved in the conformational rearrangement of the mGlu7 VFTs. The red arrows indicate the movement of the mGlu7 VFT in the ELFN1-bound structure relative to the ELFN1-free structure. (G) Extra hydrophilic contacts between the two receptor subunits in the ELFN1-bound structure compared to the ELFN1-free inactive structure.

The trFRET assay was also used to assess the importance of the glycosylation in ELFN1. The N59Q mutant of ELFN1^ECD^ exhibited a similar effect on the reduction of the trFRET signal of mGlu7 to that of the wild-type ELFN1^ECD^, whereas N90Q abolished such effect on the VFT conformational changes of the receptor (Figs. 2A and S4D; Table S2). These data suggest that the glycosylation at N90 plays a more important role in the recognition between ELFN1 and mGlu7, which could be explained by the mGlu7-ELFN1 binding mode that the N90-associated glycans form a more compact interaction with the receptor.

We further monitored the glutamate-induced G_i_ protein activation of mGlu7 using a bioluminescence resonance energy transfer (BRET) assay. It was observed that addition of the ELFN1^ECD^ protein (2 μmol/L) increased the maximal response (*E*_max_) of the glutamate-induced G_i_ activation by ∼40% for the wild-type receptor (Fig. 2B and 2C; Table S3), which suggests that ELFN1 acts as a positive allosteric modulator (PAM) to promote the glutamate-stimulated receptor activation of mGlu7. Structural alignment of the ELFN1-free, inactive mGlu7 (PDB ID: 7EPC) and mGlu7–ELFN1^ECD^ complexes revealed a compaction of the VFT dimer in the ELFN1-bound receptor (Fig. S3A–D and Video S1). The ELFN1^ECD^ binding induces a downward movement of helix L and its neighboring loops in the mGlu7 VFT, which makes the two VFTs closer by introducing extra inter-subunit interactions (Fig. 2D and Video S1). In conformation A of the mGlu7–2 × ELFN1^ECD^ complex, the ELFN1 residue W30 pushes F364 and A365 in helix L of mGlu7 towards the agonist binding cleft with the Cα atom of A365 moving by 5 Å compared to that in the ELFN1-free, inactive mGlu7 (Fig. 2E). Subsequently, helix C and the connecting loop between helix D and β-sheet f are pushed towards their counterparts in the neighboring subunit through a hydrophobic interaction network formed by the residues W363, L79, F55, L54, and V165, which leads to the introduction of interactions between R170 in helix C and R191 in the two mGlu7 subunits (Fig. 2F and 2G). The alanine substitution of R191 abolishes the glutamate-induced G_i_ activation of mGlu7, which supports the importance of this residue in mGlu7 activation (Fig. S4E). It has been demonstrated that rotation of helices B and C occurs during mGlu activation (Koehl et al., 2019). The close proximity between R191 and helix C in the two receptor subunits of the mGlu7–ELFN1^ECD^ complexes potentially facilitates the conformational rearrangement of the receptor VFT that is required for receptor activation (Lin et al., 2021) (Fig. 2G). Thus, it is likely that ELFN1 modulates the receptor activity by altering the inter-subunit interaction pattern (Fig. S3H).

To identify the critical sites for regulation, alanine mutations were introduced to the key residues in the ELFN1 binding pocket. The alanine or tryptophan substitutions of the mGlu7 residues E356, R359, A365, L376, T377, I378, S379, and K389 impair the ELFN1-mediated *E_max_* increment of the glutamate-induced G_i_ protein activation (Figs. 2C and S4F–M; Table S3), which suggests key roles of these residues in regulating receptor function. The nanobody Nb43, a PAM of mGlu5 (Koehl et al., 2019), binds to mGlu5 in a similar site to that of ELFN1 in mGlu7, where the complementarity-determining regions 2 and 3 (CDR2 and CDR3) of Nb43 clamp the L-M loop (residues 368–373) in the mGlu5 VFTs (Koehl et al., 2019) (Fig. S3I). This, together with our mGlu7–ELFN1^ECD^ structures, implies that the cleft surrounded by helix L and the neighboring loops at the apex of the mGlu VFT may serve as a potential modulatory site for this receptor family.

Given the central role of the ELFN1 N terminus in regulating the mGlu7 activity, an ELFN1-derived peptide (ELFN1^D28-Q48^) was tested for its effect on the glutamate-induced G_i_ activation of mGlu7. The data show that in the presence of 10 μmol/L peptide, the *E*_max_ was increased by ∼70% for the wild-type mGlu7 (Fig. 2B and Table S3), which indicates that the N-terminal peptide of ELFN1 is sufficient to recognize and modulate receptor function, and could serve as a novel modulator for mGlu7. Further optimization of this ELFN1 peptide would be beneficial to the drug discovery of this receptor family.

A previous study using pull-down assays suggests that ELFN1 selectively interacts with group III mGlus but not group I and II receptors (Dunn et al., 2018). Sequence alignment reveals that the key residues within the major ELFN1 binding pocket that accommodates the ELFN1 residue W30 are conserved among the group III mGlus but vary in the other mGlus (Fig. S5). The residues R359 and A365 are substituted by S/T/H and R/P/Q, respectively, in group I and group II receptors (Fig. S5). The mutations R359S and R359T in mGlu7, which disturb the π-cation interaction with the ELFN1 residue W30, and the mutations A365R and A365Q, which potentially introduce a steric hindrance to block ELFN1 binding, substantially reduced the ELFN1^ECD^ protein-associated increment of glutamate-induced G_i_ activation for the wild-type mGlu7 (Figs. 2C and S4N–Q; Table S3).

Collectively, this study provides molecular insights into the transsynaptic interaction and functional modulation between mGlu7 and ELFN1. The ELFN1 binding site at the apex of the receptor serves as an allosteric modulation site that may potentially benefit development of novel therapeutic agents.

## Supplementary Material

pwag034_Supplementary_Data

## Footnotes

The cryo-EM studies were performed at the electron microscopy facility of Shanghai Institute of Materia Medica (SIMM), Chinese Academy of Sciences. We thank M. Wang from SIMM for cryo-EM data collection. B.W. is a SANS Exploration Scholar.

This work was supported by the National Key R&D Program of China 2022YFA1302900 (B.W. and S.H.), CAS Project for Young Scientists in Basic Research YSBR-106 (B.W., Q.Z., and S.L.), CAS Strategic Priority Research Programs XDB0830000 (B.W.) and XDB1060000 (Q.Z.), National Science Foundation of China grants 32530052 (B.W.), 32400997 (S.L.), and 82121005 (B.W.), and Shanghai Pilot Program for Basic Research—Chinese Academy of Sciences, Shanghai Branch JCYJ-SHFY-2021-008 (B.W.).

The authors declare that they have no competing interests and their agreement to participate. Authors give their consent for publication.

Atomic coordinates and cryo-EM density maps for the structures of mGlu7–2 × ELFN1^ECD^ in conformations A-D and mGlu7–1 × ELFN1^ECD^ have been deposited in the Protein Data Bank (PDB) under identification codes 25UJ, 25TY, 25TX, 25TW, and 25TV, and in the Electron Microscopy Data Bank under accession code EMD-80380, EMD-80376, EMD-80375, EMD-80374, and EMD-80373, respectively. All other data will be made available upon request. Correspondence and material requests should be addressed to Shuling Lin (linshuling@simm.ac.cn), Qiang Zhao (zhaoq@simm.ac.cn) and Beili Wu (beiliwu@simm.ac.cn).

S.L. designed experiments, analyzed data, collected cryo-EM data, performed cryo-EM data processing and analysis, model building and structure refinement, and wrote the first draft. Y.F. prepared protein samples for cryo-EM studies, collected cryo-EM data, performed cryo-EM data processing and analysis, performed functional assays, and helped with manuscript preparation. S.S. performed functional assays and helped with data analysis and manuscript preparation. M.C. helped with protein sample preparation. X.C. and C.Y. expressed the proteins. S.H. helped with structure refinement and data analysis. Q.Z. and B.W. initiated the project, planned and analyzed experiments, supervised the research, and wrote the manuscript with input from all co-authors.

No AI-assisted technologies were used in this work.
