# Supplementary material for: Structural basis for the synaptic adhesion protein ELFN1-mediated modulation of mGlu7
Source: Protein Cell. 2026 May 22;17(11):1072–7. doi: 10.1093/procel/pwag034 (PMC13721118; doi:10.1093/procel/pwag034)
Supplement: Supplementary file 1 [file pwag034_supplementary_data.zip › Wu_supplementary_materials/Wu supplementary materials_new.pdf]

Supplementary Materials for

Structural basis for the synaptic adhesion protein ELFN1-mediated modulation of mGlu7

Shuling Lin[1,‡,*], Ye Feng[2, ‡], Shanhui Shui[1,3,‡], Mengmeng Chen[4], Xiaojing Chu[1], Cuiying Yi[1], Shuo Han[1,3], Qiang Zhao[1,3,5,*], Beili Wu[1,2,3,4,6,*]

[1]State Key Laboratory of Drug Research, State Key Laboratory of Chemical Biology, Shanghai Institute of Materia Medica, Chinese Academy of Sciences, Shanghai, China.

[2]School of Life Science and Technology, ShanghaiTech University, Shanghai, China.

[3]University of Chinese Academy of Sciences, Beijing, China.

[4]School of Pharmaceutical Science and Technology, Hangzhou Institute for Advanced Study, University of Chinese Academy of Sciences, Hangzhou, China.

[5]Zhongshan Institute for Drug Discovery, Shanghai Institute of Materia Medica, Chinese Academy of Sciences, Zhongshan, China.

[6]Shanghai Academy of Natural Sciences (SANS), Shanghai Institute of Materia Medica, Chinese Academy of Sciences, Shanghai, China.

[‡]These authors contributed equally to this work.

[*]Correspondence: linshuling@simm.ac.cn (S. Lin), zhaoq@simm.ac.cn (Q. Zhao), beiliwu@simm.ac.cn (B. Wu)

This PDF file includes:

Materials and Methods

Figures S1 to S5

Tables S1 to S3

Video S1 legend

References

Materials and Methods

Construct cloning and protein expression

To facilitate protein expression, the human *GRM7* gene was cloned into pFastBac1 expression vector with the endogenous signal peptide replaced with a haemagglutinin (HA) signal peptide, which was followed by a Flag epitope tag, 2 × Strep tag, and a TEV protease cleavage site. To improve protein expression and stability, the ectodomain of human *ELFN1* gene (residues 28-418) was cloned into pFastBac1 expression vector with the prolactin signal peptide at the N terminus and a 10×His tag following a PreScission protease (PPase) cleavage site at the C terminus.

The mGlu7 was expressed in High Five cells using Bac-to-Bac Baculovirus Expression System (Invitrogen). The cells were grown to a density of 1.5×10^6 cells per ml and were infected with viral stock at the multiplicity of infection of 5. The cells were cultured at 27 °C for 48 h and then collected by centrifugation at 2,000*g* for 15 min. The biomass was stored at –80 °C until use. The modified ELFN1 ectodomain was expressed using the same protocol. When the cells were harvested by centrifugation, the supernatant was kept for protein purification.

Purification of ELFN1^{ECD}

The supernatant of cell culture expressing ELFN1 ectodomain was collected by centrifugation at 2,000*g* for 15 min and filtered by 0.22 μm membrane filter (Millipore) to discard cell debris. The supernatant was then incubated with TALON resin (Clontech) and 20 mM imidazole at 4 °C overnight. The resin was then washed with 20 column volumes (CVs) of buffer containing 25 mM HEPES, pH 7.5, 150 mM NaCl, and 50 mM imidazole. The His-tagged ELFN1 ectodomain protein was eluted with 5 column volumes (CVs) of buffer containing 25 mM

HEPES, pH 7.5, 150 mM NaCl and 300 mM imidazole. The elution was concentrated and supplemented with 20% (v/v) glycerol. The protein was flash frozen and stored at –80 °C until use.

Purification of the mGlu7–ELFN1^{ECD} complex

The cells expressing mGlu7 were suspended in a buffer containing 25 mM HEPES, pH 7.5, 50 mM NaCl and EDTA-free protease cocktail inhibitor (Roche). The suspension was ultracentrifuged at 100,000*g* for 30 min. The membrane was then solubilized in 25 mM HEPES, pH 7.5, 1% (w/v) n-dodecyl-β-D-maltopyranoside (DDM, Anatrace), 0.2% (w/v) cholesterol hemisuccinate (CHS, Sigma) and 150 mM NaCl at 4 °C for 3 h. The supernatant was collected by centrifugation at 100,000*g* for 30 min and incubated with Strep resin (IBA) at 4 °C overnight. After incubation, the Strep resin was collected and washed with 10 CVs of buffer A containing 0.05% (w/v) DDM, 0.01% (w/v) CHS, 150 mM NaCl and 25 mM HEPES, pH 7.5. The resin was then incubated with 1.5 molar excess his-tagged ELFN1 ectodomain protein in a buffer containing 25 mM HEPES, pH 7.5, 150 mM NaCl, 0.25% (w/v) glyco-diosgenin (GDN, Anatrace) at 4 °C for 1.5 h to assemble the ELFN1–mGlu7 complex and exchange the detergent. After incubation, the Strep resin was washed with 20 CVs of buffer B containing 25 mM HEPES, pH 7.5, 150 mM NaCl and 0.01% (w/v) GDN. The protein was eluted with a buffer containing 200 mM Tris, pH 8.0, 150 mM NaCl, 50 mM biotin and 0.01% (w/v) GDN. To further purify the mGlu7–ELFN1^{ECD} complex, the elution was incubated with Ni-NTA resin at 4 °C for 1.5 h. The resin was washed with buffer B supplemented with 30 mM imidazole and then eluted with buffer B supplement with 300 mM imidazole. The elution was concentrated to 6 mg ml^{-1} using a 100-kDa molecular weight cutoff concentrator (Millipore) for the cryo-EM experiments.

Cryo-EM data collection and processing

3 μL of the mGlu7–ELFN1^{ECD} complex protein was applied to glow-discharged holey grid (ANTcryo R1.2/1.3, Au 300 mesh) at 4 °C and 100% humidity. The excess sample was blotted away with blot force of 1 and blot time for 3 s. The grids were subsequently flash-frozen in liquid ethane cooled by liquid nitrogen using a Mark IV Vitrobot (Thermo Fisher Scientific). The cryo-EM data were collected on a 300 kV Titan Krios G3 electron microscope (FEI) equipped with a Gatan K3 Summit direct electron detector and GIF-Quantum energy filter. The movies were recorded at a magnification of 81,000× with a bin2 pixel size of 1.071 Å using the super-resolution counting mode of SerialEM (Mastronarde, 2005). The defocus values of movies ranged from −0.8 to −1.5 μm, and the total exposure time was 3 s for 40 frames with the dose rate of 1.75 electrons per Å^2 per frame.

Cryo-EM data processing and model building

A total of 5,422 movies were collected and subjected to motion correction using MotionCor2 (Zheng et al., 2017) and the contrast transfer function (CTF) parameters of each micrograph were determined by CTFFIND4 in CryoSPARC (Punjani et al., 2017). The following data processing procedures were performed by CryoSPARC, except that the Bayesian polishing were conducted in RELION-3.1 (Zivanov et al., 2018). 2,316,786 particles were auto-picked using the blob picker and down-sampled by 4 × binning. The particles were subsequently subjected to several rounds of 2D classification to remove the false-positive particles. The best-looking classes of 1,341,279 particles were subjected to ab-initio reconstruction and heterogeneous refinement in C1 symmetry. Two best-looking classes of 839,346 particles were combined and subjected to non-uniform refinement (C1, Bin1) and Bayesian polishing to improve the resolution. The polished particles were further subjected to 3D classification generating five classes of particles with either one or two molecule(s) of the ELFN1 ectodomain bound (termed mGlu7–1×ELFN1^{ECD} and mGlu7–2×ELFN1^{ECD}), which were

subjected for further processing. The best-looking classes of mGlu7–2×ELFN1^{ECD} of 337,285 particles were subjected to non-uniform refinement (C2) and local refinement with the mask on the ECD of mGlu7 and the visible part of ELFN1^{ECD}, generating a final map at the resolution of 3.1 Å (conformation A).

For the mGlu7–2×ELFN1^{ECD} class with discernable densities of the mGlu7 TMDs (conformation B), 168,491 particles were subjected to non-uniform refinement (C2) and local refinement with the mask on the ECD of mGlu7 and the visible part of ELFN1^{ECD}, generating a map with the resolution at 3.6 Å. For the mGlu7–2×ELFN1^{ECD} class with conformational heterogeneous (conformation D), 151,592 particles were subjected to non-uniform refinement (C1), resulting in a map with the resolution at 3.5 Å. For the mGlu7–1×ELFN1^{ECD} class, 173,226 particles were further subjected to 2D classification, ab-initio reconstruction and heterogeneous refinement, generating one class of blurry particles and two classes of particles showing one or two molecules of ELFN1^{ECD}. 46,831 particles containing two molecules of ELFN1^{ECD} (conformation C) were subjected to non-uniform refinement (C1), resulting in a map at 4.0 Å resolution. 64,331 particles with only one molecule of ELFN^{ECD} were subjected to non-uniform refinement (C1) and local refinement using a mask encompassing the ECD of mGlu7 and visible part of ELFN1^{ECD}, resulting in a final map with a global resolution at 3.8 Å (mGlu7–1×ELFN1^{ECD}). The resolutions of all density maps were calculated using gold-standard Fourier shell correlation (FSC) with the 0.143 criterion.

The initial models of the mGlu7–ELFN1^{ECD} complexes were built by docking the ECDs of mGlu7 from the full-length inactive mGlu7 structure (PDB ID: 7EPC) and the ELFN1^{ECD} generated by AlphaFold server (Jumper et al., 2021) into the maps using ChimeraX (Goddard et al., 2018). The models were manually adjusted in Coot (Emsley and Cowtan, 2004) and refined by several rounds of real-space refinement in PHENIX (Adams et al., 2011). The final

models were validated using MolProbity (Williams et al., 2018). The figures were prepared using ChimeraX or pymol.

Time-resolved FRET (trFRET) Assay

The trFRET assays were conducted to investigate the effect of ELFN1 on the conformational changes of the mGlu7 VFTs. Human *GRM7* (H41-I915) gene was cloned into a pTT5 vector with an HA signal peptide, followed by a Flag tag and a SNAP tag at the N terminus. HEK293T cells (Invitrogen) were seeded at a density of 2×10^5 cells per well in 6-well plates and incubated at 37 °C with 5% CO_2 one day before transfection. The cells were co-transfected with 1 μg plasmids of the SNAP-tagged mGlu7 and 200 ng plasmids of the glutamate transporter EAAT1 and incubated at 37 °C with 5% CO_2.

After 48 h post transfection, the cells were detached with 0.25% trypsin-EDTA and resuspend in assay buffer (1× Hanks' balanced salt solution (HBSS) supplemented with 20 mM HEPES, pH 7.4; Gibco). For FRET labelling, the cells were incubated with 100 nM SNAP-Lumi4-Tb (donor fluorophore; PerkinElmer) and 60 nM SNAP-Green (acceptor fluorophore; PerkinElmer) in Tag-lite buffer (PerkinElmer) at 37 °C for 1 h. The cells were then washed three times and resuspended with assay buffer, the ELFN1 ectodomain protein (or the substitutions) was added to the cell suspension at a final concentration of 0.7 μM. 20,000 cells in 10 μl were dispensed per well into 384-well shallow microplates (PerkinElmer). After incubation at 37 °C for 15 min, 10 μl of glutamate diluted in the assay buffer at different concentrations (100 mM–1.5 μM) were added to each well. trFRET signals were measured using a Synergy II plate reader (BioTek). Fluorescence excitation was performed at 340 nm, and emission at 520 nm was collected in two time-resolved windows: 50–100 μs (Window 1) and 1200–1600 μs (Window 2). The FRET signal was expressed as the ratio of fluorescence intensities in Window 1 to Window 2. Changes in this ratio between the ELFN1-treated and -

untreated samples were used to assess the ELFN1-induced modulation of the mGlu7 conformational changes. All data were analyzed in GraphPad Prism 9.0.

BRET assay using TRUPATH biosensor

To evaluate the regulatory effect of ELFN1 and its modified peptide on the G_i protein activation of mGlu7 and mGlu8, BRET assay was conducted using TRUPATH biosensors (Olsen et al., 2020), which monitor the interaction between the Gα and Gγ subunits. The *GRM7* (or mutants) gene was cloned into a pTT5 vector with an N-terminal HA signal peptide and a Flag epitope. The HEK293F cells (Invitrogen) at a density of 1.5×10^6 cells ml^{-1} were co-transfected with plasmids of mGlu7, $G\alpha_{i1}$-Rluc8, $G\beta_3$, $G\gamma_9$-GFP2 (Addgene kit no. 1000000163) and the glutamate transporter EAAT1 at the final plasmid concentration of 1 μg mL^{-1} (plasmid ratio: 4:1:1:1:1). The cells were cultivated in 5% CO_2 at 37 °C for 48 h. Surface expression of Flag-tagged receptors was measured using PE-conjugated anti-Flag antibody (ABclonal) and the fluorescent signal was measured by applying compensation to subtract the GFP signal using a Guava easyCyte flow cytometry (Luminex).

To monitor ELFN1-mediated modulation, the cells were resuspended in BRET2 buffer (1× HBSS supplemented with 20 mM HEPES, pH 7.4) supplemented with 2 μM recombinant $ELFN1^{ECD}$ protein, 10 μM synthesized peptide derived from the residues D28-Q48 in the ELFN1 N terminus (dissolved in 1 × PBS), or the buffer in the final purified $ELFN1^{ECD}$ protein sample. Then, the cells were added into white 96-well plates (Beyotime Biotechnology) at a density of 60,000 cells per well in a final volume of 60 μl. After incubation at 37 °C for 30 min, 10 μl of freshly prepared coelenterazine 400a (Nanolight Technologies) was added into the plate at the final concentration of 50 μM, followed by 5 minutes equilibration in the dark. The baseline was measured four times in 16 min using a Synergy II plate reader (BioTek) with 410 nm (Rluc8-coelenterazine) and 515 nm (GFP2) emission filters. Then, 30 μL of glutamate at

different concentrations (5 nM to 50 mM) were added into each well, and the signals were measured five times in 20 min. The BRET ratios were calculated as the ratio of the GFP2 emission to Rluc8-coelenterazine emission. All the BRET data were analyzed using GraphPad Prism 9.0.

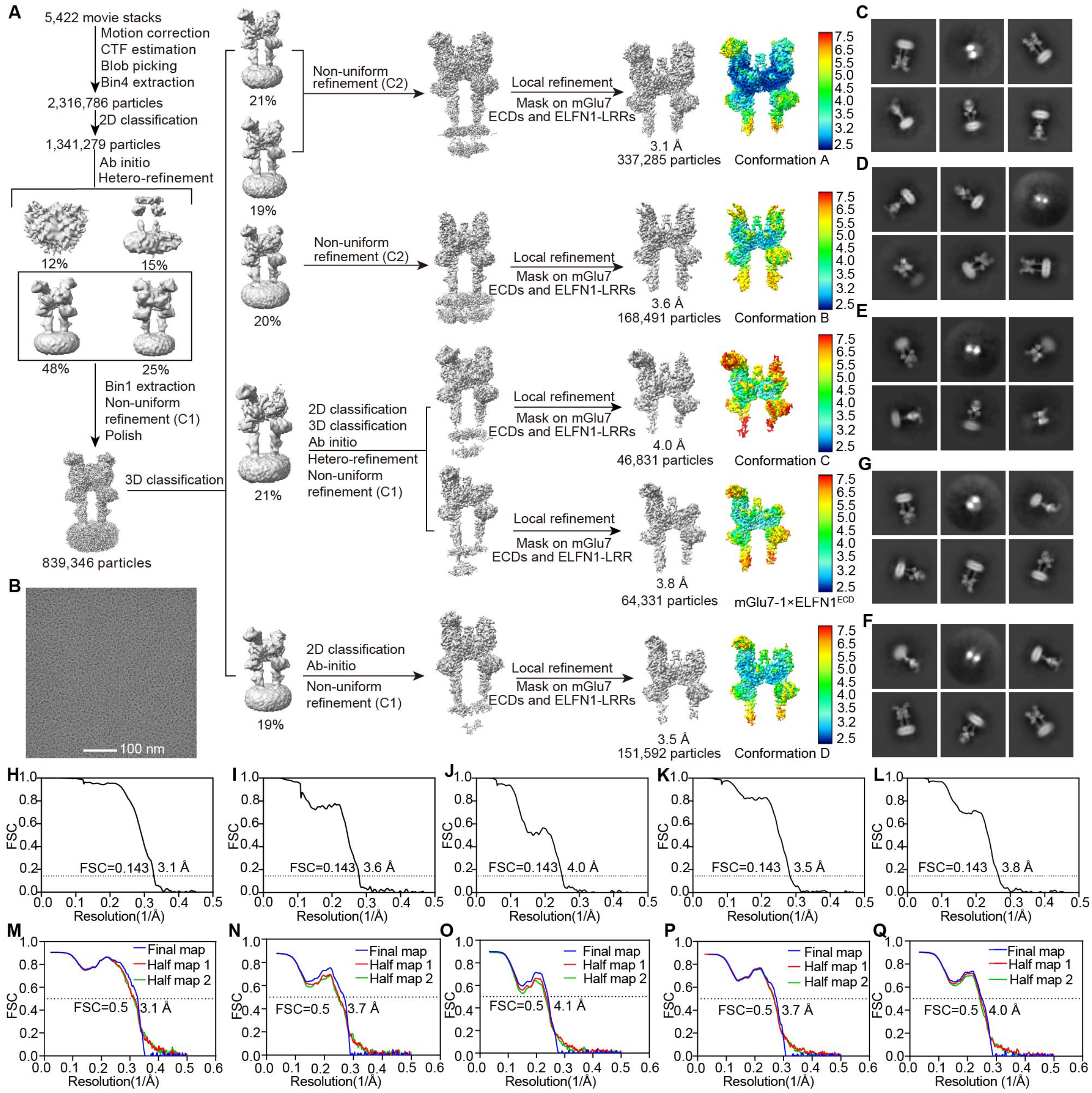

Figure S1. Cryo-EM data processing and 3D reconstruction workflow. (A) Data processing workflow, with cryo-EM maps colored according to local resolution (in Å). (B) Representative cryo-EM image from two independent experiments. (C-G) 2D averages of mGlu7–2×ELFN1^{ECD} in conformations A-D (C-F) and mGlu7–1×ELFN1^{ECD} (G). (H-L) Gold-standard Fourier shell correlation (FSC) curves of mGlu7–2×ELFN1^{ECD} in conformations A-D (H-K) and mGlu7–1×ELFN1^{ECD} (L). (M-Q) Cross-validation of model to cryo-EM density map for mGlu7–2×ELFN1^{ECD} in conformations A-D (M-P) and mGlu7–1×ELFN1^{ECD} (Q). FSC curves for the final model versus the final map and half maps are shown in blue, red and green,

respectively.

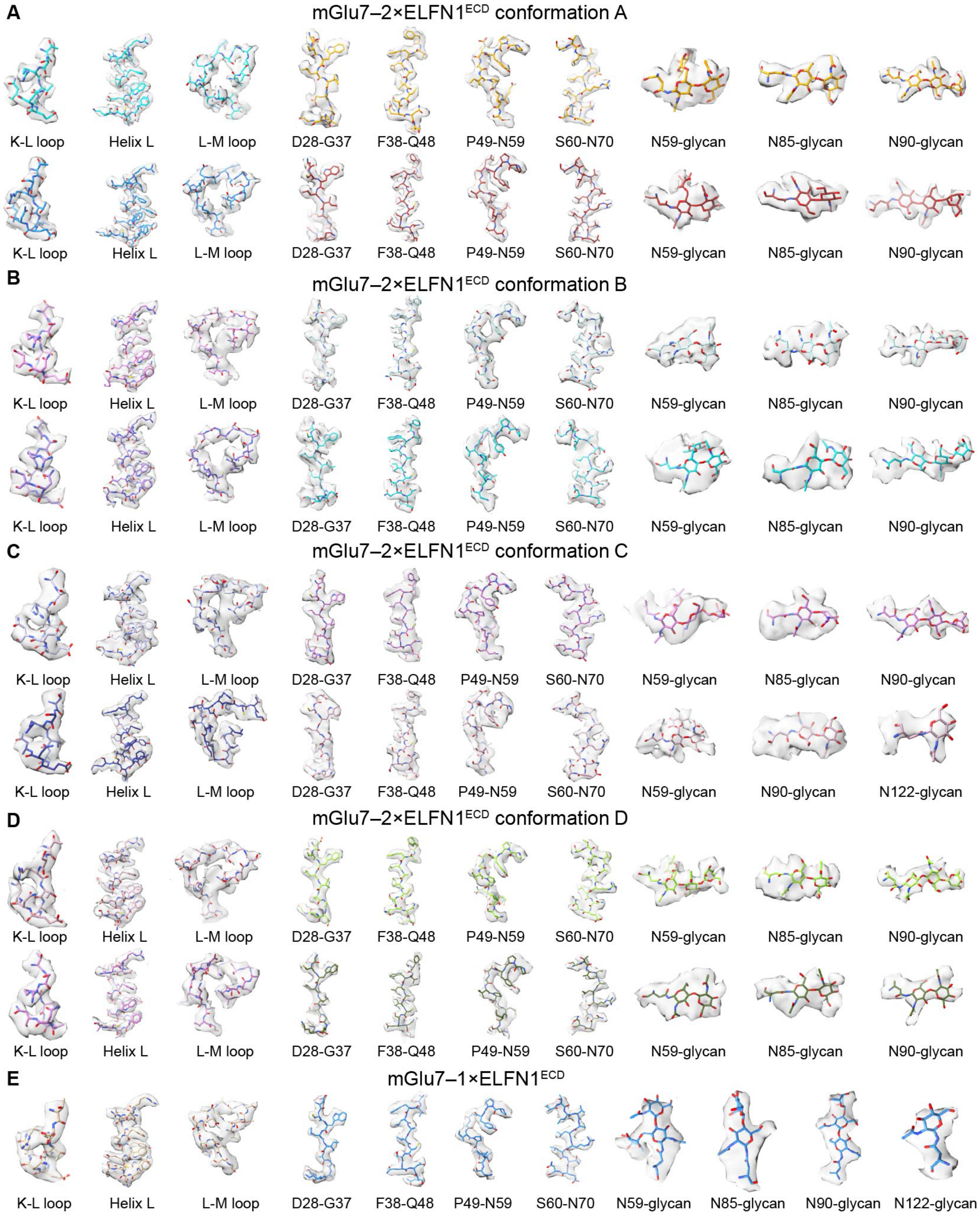

Figure S2. Cryo-EM density maps of mGlu7–2×ELFN1^{ECD} in conformations A-D and mGlu7–1×ELFN1^{ECD}. Cryo-EM maps and models of the five structures are shown for helix L and its connecting loops (K-L and L-M loops) of the receptor, and the N-terminal regions (D28-N70) and glycosylation of ELFN1. The models are shown as sticks. The maps are colored grey.

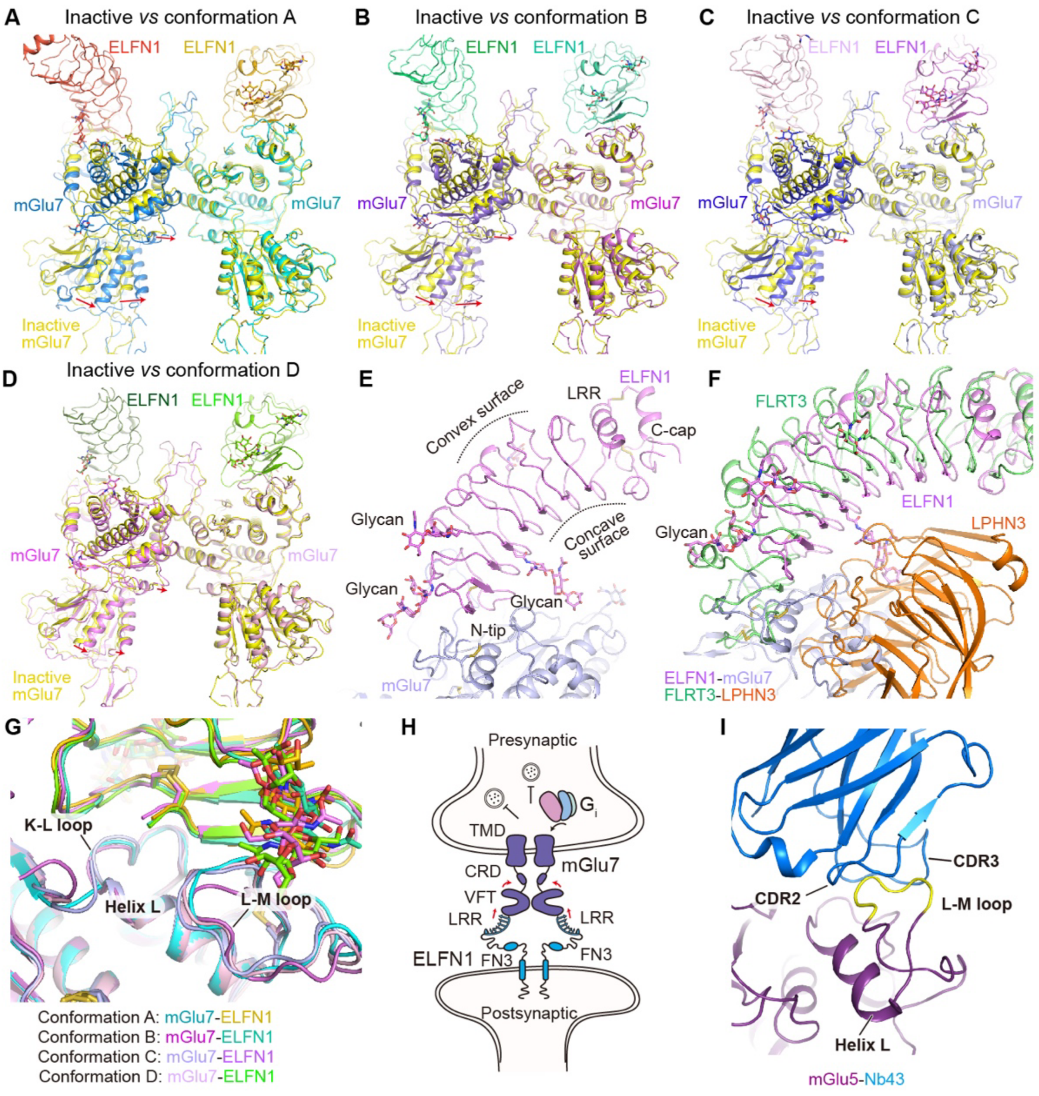

Figure S3. Structural comparison of the mGlu structures. (A-D) Structural comparison of the mGlu7–2×ELFN1^{ECD} complexes and inactive mGlu7 (PDB ID: 7EPC). (E) Overall conformation of ELFN1-LRR in the mGlu7–ELFN1^{ECD} complex. (F) Comparison of the mGlu7–ELFN1^{ECD} structure and the structure of the LRR domain in the fibronectin leucine rich transmembrane protein 3 (FLRT3) in complex with latrophilin-3 (LPHN3) olfactomedin domain (PDB ID: 5CMN) (Lu et al., 2015). (G) Structural comparison of the ELFN1 binding site in conformations A-D of the mGlu7–2×ELFN1^{ECD} complex. (H) Schematic representation illustrating the trans-synaptic interaction and modulation between mGlu7 and ELFN1. The conformational changes are indicated by red arrows. G protein coupling is indicated by a black arrow. ELFN1 anchors mGlu7 in specific subsynaptic localization and modulates its function.

Activation of mGlu7 triggers intracellular G_i signaling and depresses the vesicle release. (I) Binding interface between mGlu5 and its nanobody Nb43 (PDB ID: 6N51).

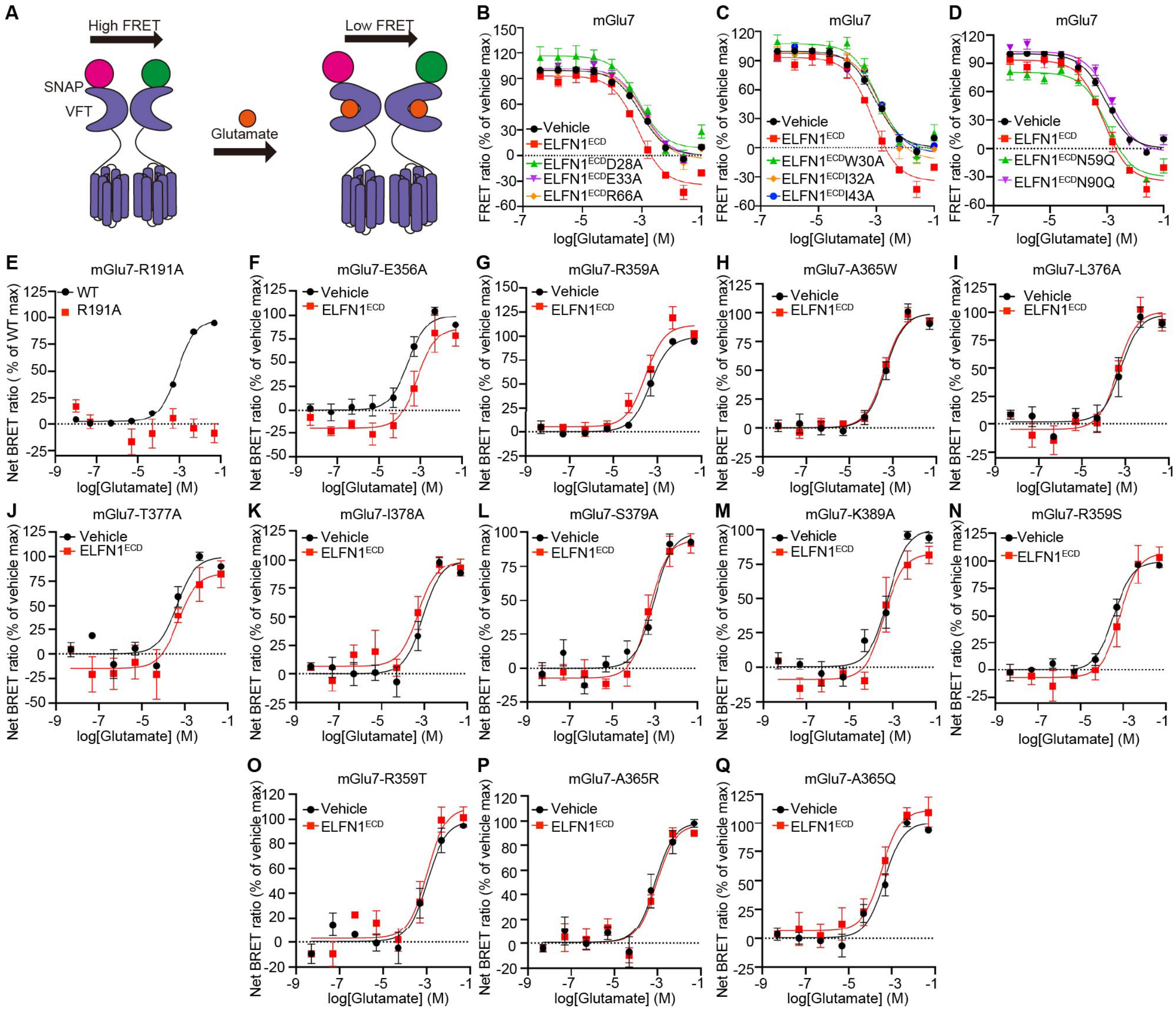

Figure S4. trFRET and BRET assays of the mGlus. (A) Schematic illustration of the trFRET assay of fluorophore-labelled mGlu7 with a transition from high FRET signal to low FRER signal upon glutamate stimulation. (B-D) Glutamate-induced conformational changes of the mGlu7 VFTs monitored by trFRET assays in the absence (vehicle) or presence of $ELFN1^{ECD}$ or mutants. Data are shown as mean ± s.e.m. from at least three independent experiments performed in technical triplicate. Table S2 provides detailed numbers of independent experiments (n) and statistical evaluation. (E) Glutamate-induced G_i activation of the wild-type mGlu7 and mutant measured by BRET assays. Data are shown as mean ± s.e.m. from at least three independent experiments performed in technical duplicate. Numbers of independent

experiments (n): WT, n = 58; R191A, n = 3. Cell surface expression levels (% of WT, $n \geqslant 3$): R191A, 87 ± 1. (F-Q) Glutamate-induced G_i activation of the wild-type mGlu7 and its mutants in the absence (vehicle) or presence of $ELFN1^{ECD}$ measured by BRET assays. Data are shown as mean ± s.e.m. from at least three independent experiments performed in technical duplicate. Tables S3 provide detailed numbers of independent experiments (n) and statistical evaluation.

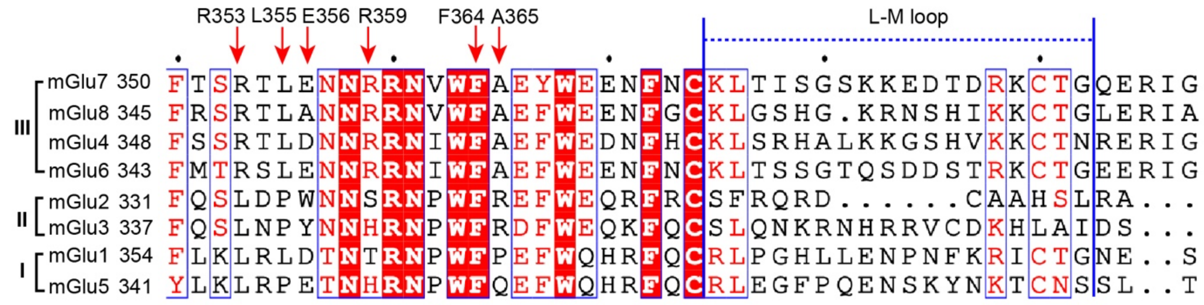

Fig. S5 Sequence alignment of the mGlus. The residues within the ELFN1^{ECD} binding pocket are highlighted by red arrows. The alignment was generated using UniProt (UniProt, 2025) and the graphic was prepared on the ESPript 3.0 server (Robert et al., 2025).

Table S1 Cryo-EM data collection, refinement and validation statistics

	mGlu7–2×ELFN1^{ECD}				
	Conformation A (EMDB-80380) (PDB 25UJ)	Conformation B (EMDB-80376) (PDB 25TY)	Conformation C (EMDB-80375) (PDB 25TX)	Conformation D (EMDB-80374) (PDB 25TW)	mGlu7–1×ELFN1 (EMDB-80373) (PDB 25TV)
**Data collection and processing**					
Magnification	81,000	81,000	81,000	81,000	81,000
Voltage (kV)	300	300	300	300	300
Electron exposure ($e^-/Å^2$)	70	70	70	70	70
Defocus range (μm)	–0.8 ~ –1.5	–0.8 ~ –1.5	–0.8 ~ –1.5	–0.8 ~ –1.5	–0.8 ~ –1.5
Pixel size (Å)	1.071	1.071	1.071	1.071	1.071
Symmetry imposed	C2	C2	C1	C1	C1
Initial particle images (no.)	2,316,786	2,316,786	2,316,786	2,316,786	2,316,786
Final particle images (no.)	337,285	168,491	46,831	151,592	64,331
Map resolution (Å)	3.1	3.6	4.0	3.5	3.8
FSC threshold	0.143	0.143	0.143	0.143	0.143
Map resolution range (Å)	2.5-7.5	2.5-7.5	2.5-7.5	2.5-7.5	2.5-7.5
**Refinement**					
Initial model used (PDB code)	7EPC	7EPC	7EPC	7EPC	7EPC
Model resolution (Å)	3.1	3.7	4.1	3.7	4.0
FSC threshold	0.5	0.5	0.5	0.5	0.5
Map sharpening *B* factor ($Å^2$)	-92	-88	-71	-87	-71
Model composition					
Non-hydrogen atoms	10,315	10,289	10,500	10,315	9314
Protein residues	1353	1360	1382	1358	1247
B factors ($Å^2$)					
Protein	47.80	83.86	69.61	28.31	50.90
R.m.s. deviations					
Bond lengths (Å)	0.002	0.002	0.002	0.002	0.002
Bond angles (°)	0.404	0.534	0.490	0.434	0.509
Validation					
MolProbity score	1.74	1.67	1.77	1.67	1.79
Clashscore	7.30	6.46	7.22	7.78	8.15
Poor rotamers (%)	0.10	0.80	0.00	0.10	0.34
Ramachandran plot (%)					
Favored	95.06	95.46	94.49	96.33	94.96
Allowed	4.94	4.54	5.51	3.67	5.04
Disallowed	0.00	0.00	0.00	0.00	0.00

Table S2 Glutamate-induced conformational changes of the mGlu7 VFTs monitored by trFRET assays

Mutants[a]	EC_{50} (mM)	pEC_{50} (mean ± s.e.m.)[b]	Span[c] (% of mGlu7)[b]	$FRET_{basal}$[d] (% of mGlu7)[b]	$FRET_h$[e] (% of mGlu7 $FRET_{basal}$)[b]	n[f]
mGlu7	1.0	3.05 ± 0.06	100 ± 3	100 ± 2	0 ± 2	11
mGlu7 + $ELFN1^{ECD}$	1.0	3.12 ± 0.10	128 ± 6***	94 ± 4	-35 ± 5***	5
mGlu7 + $ELFN1^{D28A}$	1.0	3.09 ± 0.14	109 ± 7	117 ± 4**	8 ± 6	5
mGlu7 + $ELFN1^{W30A}$	1.0	2.96 ± 0.11	110 ± 6	108 ± 3	-3 ± 5	4
mGlu7 + $ELFN1^{I32A}$	1.0	2.84 ± 0.09	111 ± 5	98 ± 3	-12 ± 4	3
mGlu7 + $ELFN1^{E33A}$	1.0	2.94 ± 0.07	102 ± 4	102 ± 2	0 ± 3	4
mGlu7 + $ELFN1^{I43A}$	1.0	2.87 ± 0.09	103 ± 4	98 ± 3	-5 ± 4	3
mGlu7 + $ELFN1^{R66A}$	1.0	2.92 ± 0.09	104 ± 4	100 ± 3	-4 ± 4	3
mGlu7 + $ELFN1^{N59Q}$	1.0	3.00 ± 0.10	111 ± 5	80 ± 3**	-30 ± 4***	3
mGlu7 + $ELFN1^{N90Q}$	1.0	2.87 ± 0.10	105 ± 5	102 ± 3	-3 ± 4	3

[a]All mutations were introduced in the wild-type $ELFN1^{ECD}$.

[b]Data are mean ± s.e.m. from at least three independent experiments. $**P<0.001$, $***P<0.0001$ by one-way analysis of variance followed by Dunnett's post-test compared to the response of mGlu7.

[c]The span is defined as the window between the maximal glutamate response and the basal trFRET signal (glutamate-free).

[d]$FRET_{basal}$, the FRET signal monitored in the absence of glutamate.

[e]$FRET_h$, the FRET signal monitored in the presence of glutamate at the highest concentration tested in this study.

[f]Sample size, the number of independent experiments performed in technical triplicate.

Table S3 Glutamate-induced G_i activation of mGlu7 and its mutants in the absence or presence of ELFN1^{ECD} measured by BRET assays

Mutants[a]	EC_{50} (mM)	pEC_{50} (mean ± s.e.m.)[b]	E_{max}[c] (% of vehicle)[c]	n[d]
mGlu7 WT	0.80	3.10 ± 0.04	100 ± 2	58
WT + ELFN1^{ECD}	0.93	3.03 ± 0.06	138 ± 4***	35
WT + ELFN1$^{D28\text{-}Q28}$	1.5	2.67 ± 0.21*	170 ± 20***	6
E356A	0.25	3.60 ± 0.16	100 ± 7	3
E356A + ELFN1^{ECD}	0.70	3.16 ± 0.23	110 ± 10	3
R359A	0.47	3.32 ± 0.10	100 ± 5	3
R359A + ELFN1^{ECD}	0.29	3.54 ± 0.17	107 ± 8	3
R359S	0.37	3.44 ± 0.09	100 ± 4	3
R359S + ELFN1^{ECD}	0.70	3.15 ± 0.19	120 ± 10	3
R359T	1.1	2.97 ± 0.19	100 ± 9	3
R359T + ELFN1^{ECD}	1.1	2.98 ± 0.22	110 ± 10	3
A365W	0.46	3.34 ± 0.12	100 ± 6	3
A365W + ELFN1^{ECD}	0.40	3.40 ± 0.10	99 ± 5	3
A365Q	0.46	3.34 ± 0.14	100 ± 6	3
A365Q + ELFN1^{ECD}	0.34	3.47 ± 0.19	105 ± 9	3
A365R	0.78	3.11 ± 0.19	100 ± 9	3
A365R + ELFN1^{ECD}	0.90	3.05 ± 0.17	98 ± 8	3
L376A	0.62	3.21 ± 0.19	100 ± 9	4
L376A + ELFN1^{ECD}	0.44	3.35 ± 0.17	110 ± 9	4
T377A	0.44	3.36 ± 0.20	100 ± 9	3
T377A + ELFN1^{ECD}	0.50	3.30 ± 0.31	100 ± 10	3
I378A	0.85	3.07 ± 0.19	100 ± 9	3
I378A + ELFN1^{ECD}	0.51	3.29 ± 0.23	90 ± 10	3
S379A	0.92	3.04 ± 0.16	100 ± 8	3
S379A + ELFN1^{ECD}	0.56	3.25 ± 0.16	103 ± 7	3
K389A	0.61	3.21 ± 0.15	100 ± 7	3
K389A + ELFN1^{ECD}	0.42	3.38 ± 0.20	92 ± 9	3

[a]All mutations were introduced in the wild-type mGlu7 (WT).

[b]Data are mean ± s.e.m. from at least three independent experiments. *P<0.05, ***P<0.0001 by two-tailed Student's t-text compared to the response of the receptor measured in the absence of ELFN1 (vehicle).

[c]The maximal response is reported as a percentage of the maximum effect at the receptor measured in the absence of ELFN1 (vehicle).

[d]Sample size, the number of independent experiments performed in technical duplicate.

Video S1 Structural morph between the ELFN1-free inactive mGlu7 and the ELFN1-bound mGlu7 (conformation A). The ELFN1^{ECD} binding induces a downward movement of helix L and its neighboring loops in the mGlu7 VFT, which makes the two VFTs closer by introducing extra inter-subunit interactions. mGlu7 and ELFN1^{ECD} are shown in cartoon representations. The key residue W30 in ELFN1 are shown in stick representations. Helix L is colored in red.

References

Adams PD, Afonine, PV, Bunkóczi, G et al. The Phenix software for automated determination of macromolecular structures. Methods 2011;**55**:94-106.

Emsley P, and Cowtan, K. Coot: model-building tools for molecular graphics. Biological crystallography 2004;**60**:2126-2132.

Goddard TD, Huang, CC, Meng, EC et al. UCSF ChimeraX: Meeting modern challenges in visualization and analysis. Protein science 2018;**27**:14-25.

Jumper J, Evans, R, Pritzel, A et al. Highly accurate protein structure prediction with AlphaFold. Nature 2021;**596**:583-589.

Lu Yue C, Nazarko, Olha V, Sando, R et al. Structural Basis of Latrophilin-FLRT-UNC5 Interaction in Cell Adhesion. Structure 2015;**23**:1678-1691.

Mastronarde DN. Automated electron microscope tomography using robust prediction of specimen movements. J Struct Biol 2005;**152**:36-51.

Olsen RH, DiBerto, JF, English, JG et al. TRUPATH, an open-source biosensor platform for interrogating the GPCR transducerome. Nature chemical biology 2020;**16**:841-849.

Punjani A, Rubinstein, JL, Fleet, DJ et al. cryoSPARC: algorithms for rapid unsupervised cryo-EM structure determination. Nat Methods 2017;**14**:290-296.

Robert X, Guillon, C, and Gouet, P. FoldScript: a web server for the efficient analysis of AI-generated 3D protein models. Nucleic Acids Res 2025;**53**:W277-W282.

UniProt C. UniProt: the Universal Protein Knowledgebase in 2025. Nucleic Acids Res 2025;**53**:D609-D617.

Williams CJ, Headd, JJ, Moriarty, NW et al. MolProbity: more and better reference data for improved all-atom structure validation. Protein Science 2018;**27**:293-315.

Zheng SQ, Palovcak, E, Armache, J-P et al. MotionCor2: anisotropic correction of beam-induced motion for improved cryo-electron microscopy. Nat. Methods 2017;**14**:331-332.

Zivanov J, Nakane, T, Forsberg, BO et al. New tools for automated high-resolution cryo-EM structure determination in RELION-3. elife 2018;**7**:e42166.
